# Separation in flowering time contributes to the maintenance of sympatric cryptic plant lineages

**DOI:** 10.1002/ece3.1481

**Published:** 2015-05-08

**Authors:** Stefan G Michalski, Walter Durka

**Affiliations:** Department of Community Ecology (BZF), Helmholtz Centre for Environmental Research UFZTheodor-Lieser-Strasse 4, Halle, D-06120, Germany

**Keywords:** Allochronic isolation, chloroplast DNA, cryptic species, haplotype sharing, hybridization, predispersal seed predation

## Abstract

Sympatric cryptic lineages are a challenge for the understanding of species coexistence and lineage diversification as well as for management, conservation, and utilization of plant genetic resources. In higher plants studies providing insights into the mechanisms creating and maintaining sympatric cryptic lineages are rare. Here, using microsatellites and chloroplast sequence data, morphometric analyses, and phenological observations, we ask whether sympatrically coexisting lineages in the common wetland plant *Juncus effusus* are ecologically differentiated and reproductively isolated. Our results show two genetically highly differentiated, homoploid lineages within *J*. *effusus* that are morphologically cryptic and have similar preference for soil moisture content. However, flowering time differed significantly between the lineages contributing to reproductive isolation and the maintenance of these lineages. Furthermore, the later flowering lineage suffered less from predispersal seed predation by a *Coleophora* moth species. Still, we detected viable and reproducing hybrids between both lineages and the earlier flowering lineage and *J*. *conglomeratus*, a coexisting close relative. Flowering time differentiation between the lineages can be explained by neutral divergence alone and together with a lack of postzygotic isolation mechanisms; the sympatric coexistence of these lineages is most likely the result of an allopatric origin with secondary contact.

## Introduction

The discovery and description of so far unrecognized biological diversity are fundamental to evolutionary biology (Hendry et al. 2010). Molecular analyses identifying lineages that are morphologically and taxonomically indistinguishable and hence are called “cryptic,” have become frequent in some taxonomic groups such as animals or fungi, but have been stated to be surprisingly rare in higher plants (Bickford et al. 2007). Obviously, the presence of cryptic lineages has major impact on management, conservation, and utilization of plant genetic resources that need to take into account the specific evolutionary history and current genetic structure (Tellier et al. 2011).

The investigation of cryptic diversity, in particular cases of sympatrically occurring cryptic lineages, is challenging because of two fundamental questions that are tightly interlinked. First, how did these lineages evolve and second, how are they ecologically and functionally maintained? Although recently cryptic diversity has been revealed by DNA bar coding in several plant species (e.g., Ragupathy et al. 2009; Ni et al. 2012; Yang et al. 2012), studies providing detailed insights into the evolutionary mechanisms and ecological causes or consequences, respectively, still remain scarce. Here, we report on sympatric cryptic lineages in a common wetland plant and ask how reproductive isolation between lineages is maintained by phenological and ecological mechanisms in the face of hybridization and putative introgression. Furthermore, we seek for indications for an allopatric or sympatric evolutionary origin of these lineages.

The diversification of evolutionary lineages and the accumulation of functional and ecological divergence among groups of individuals are facilitated by barriers reducing effective gene flow (Rieseberg and Willis 2007). However, spatial separation is not always necessary to explain the formation of reproductive isolation between groups because even in sympatry both neutral processes and divergent selection can result in population divergence (Devaux and Lande 2008; Flaxman et al. 2014). Eventually, a combination of different isolation mechanisms often leads to an almost complete separation even between closely related groups (e.g., Ramsey et al. 2003), which consequently may be accompanied by a divergence in neutral genetic variation. However, reproductive isolation between lineages is not necessarily accompanied by morphological trait divergence and thus reflected by taxonomy. The incongruence between morphology and taxonomic species boundaries in cryptic lineages has been explained by two general motives (Bickford et al. 2007). First, uniform selection may promote morphological stasis or phenotypic convergence between genetically and reproductively well-isolated lineages. Second, more recently diverged and therefore morphologically similar lineages can be genetically differentiated due to reproductive isolation. Mechanisms of reproductive isolation can be generally categorized depending upon whether they act before or after zygote formation: For example, prezygotic isolation can result from extrinsic barriers such as spatial or temporal separation (e.g., Ellis et al. 2006; Savolainen et al. 2006) or from intrinsic barriers via conspecific gamete precedence (Howard 1999; Fishman et al. 2008). Postzygotic isolation can be mediated via ovule abortion or lower hybrid fitness, which can be expressed, for example, in reduced hybrid seed weight or fertility (see examples in Levin 2012). In cryptic lineages, such isolation mechanisms can affect reproductive interaction directly via acoustical and chemical communication in animals (Henry 1994; Esselstyn et al. 2012). In higher plants, however, reproductive isolation may be driven more indirectly by pollinator preferences (Bower and Brown 2009) or by temporal separation of flowering time (Silvertown et al. 2005). Often, cryptic lineages in higher plants are the result of ploidy heterogeneity (e.g., Schönswetter et al. 2007) which can instantly lead to reproductive isolation via pre- and postzygotic barriers and which has been stated to be a major driver for speciation processes in flowering plants (Otto and Whitton 2000).

In allopatric lineages, prezygotic isolation is obvious (Borsch et al. 2011; Govindarajulu et al. 2011), and with time after divergence, additional pre- and postzygotic barriers are expected to accumulate. In sympatric lineages, the nature of reproductive isolation is often more difficult to elucidate as both pre- and postzygotic mechanisms have to be considered jointly (Whittall et al. 2004). Moreover, the selective potential of biotic interactions in a shared environment might alter the strength of potential isolating mechanisms, as, for example, shared predispersal seed predators are putatively a strong selective agent on inflorescence and flower morphology as well as the timing of reproduction (Elzinga et al. 2007). Thus, the understanding of sympatric coexistence of cryptic and possibly functionally similar lineages needs to take into account multiple nonexclusive factors such as reproductive and other biotic interactions, but also resource partitioning, microenvironmental heterogeneity, or even neutral processes (Chesson 2000; Hubbell 2005; Leibold and McPeek 2006), which all might contribute to maintain such sympatric relationships.

The common or soft rush *Juncus effusus* L. is a self-compatible, wind-pollinated species with an almost worldwide distribution and exhibits a wide ecological tolerance and morphological variability (e.g., Weimarck 1946; Agnew 1961; Kirschner et al. 2002). Because of its frequency in wetland communities and infestation potential in agriculturally used areas (e.g., McCorry and Renou 2003), *J*. *effusus* has a significant ecological as well as economic importance. Furthermore, the species has been established as a model plant in phytoremediation (e.g., Gruber et al. 2008). A preliminary molecular analysis revealed two sympatric but genetically strongly differentiated groups (S. G. Michalski, unpubl. data) giving rise to the hypothesis of cryptic lineages within the common rush. However, the evaluation of differentiation patterns in *J*. *effusus* is complicated by putative hybridization with its closest relative *J*. *conglomeratus* L. Most recent literature acknowledges the frequent occurrence of intermediate forms of putative hybrid origin (Křísa 1962; Agnew 1968; O'Mahony 2002; Wilcox 2010). Consequently, when analyzing cryptic lineages within *J*. *effusus* and their ecology, the closely related *J*. *conglomeratus* has to be included. Here, using a combination of molecular, morphometric, karyological, phenological, and ecological analyses, we test the following hypotheses:

1. In Central Europe, *Juncus effusus* consists of two genetically distinct lineages representing cryptic species. Specifically, we test whether both nuclear and plastid genomes and morphology are differentiated among lineages and their closest relative, *J*. *conglomeratus*.
2. Sympatric cryptic lineages within *J*.* effusus* are ecologically differentiated and reproductively isolated. In particular, we hypothesize that lineages (1) have different soil moisture preferences and are differently affected by predispersal seed predation and (2) are reproductively isolated by different flowering times.

A distinct ecological and functional differentiation in combination with reproductive isolation between the putative cryptic lineages and a relatively recent genetic divergence might favor the hypothesis of a sympatric origin of these coexisting lineages. On the other hand, strong genetic differentiation coupled with incomplete reproductive isolation and low ecological differentiation might indicate an allopatric origin with secondary contact leading to recent sympatric coexistence.

## Material and Methods

### Study species

The most recent taxonomic treatment of the genus *Juncus* recognizes a number of regional subspecies for *J*. *effusus* emphasizing its high morphological variability (Kirschner et al. 2002). In contrast to North America (Hämet-Ahti 1980; Zika 2003), for Central Europe, only *J*. *effusus* ssp. e*ffusus* is known. As all analyses were performed in that region, we will refer to this taxon with the shorter “*J*. *effusus”* in the following. No taxonomic units below species level are recognized for *Juncus conglomeratus* (Kirschner et al. 2002). Both species are perennial herbs growing mainly in wet, open to shady habitats. Although the species are able to reproduce vegetatively by short rhizomes, generative reproduction is likely to be more important, accomplished by the production of numerous tiny seeds with high potential for long-distance dispersal (e.g., Ervin and Wetzel 2001). *Juncus effusus* is known to be self-compatible (Edgar 1964) and like other *Juncus* species putatively predominantly selfing (Buchenau 1892; Richards and Clapham 1941b). *Juncus effusus* and *J*. *conglomeratus* have been cited to differ in a range of morphological characters, flowering time, and environmental requirements (Richards and Clapham 1941a,b; Tweed and Woodhead 1946; Agnew 1968; Kirschner et al. 2002). Additional morphometric diagnostic characters for the hybrid *J*. × *kern-reichgeltii* Jansen & Wacht. ex Reichg. have been described (O'Mahony 2002; Wilcox 2010); however, the parental species are extremely plastic in some of the diagnostic features (Agnew 1968; O'Mahony 2002). Hence, the distinction between parental species and hybrid based on morphology alone is often difficult. For European *J*. *effusus* and *J*. *conglomeratus,* only diploids are known with reported chromosome counts 2n = 40 and 42 for both species (see references in Drábková 2013).

### Sites and sampling

Our main study was conducted near Halle (Saale), Germany (51.511°N, 11.927°E) where *Juncus effusus* and *J*. *conglomeratus* co-occur on a former military training area characterized by a high edaphic heterogeneity comprising very dry as well as waterlogged areas. To assess how the genetic constitution at the main study site compares to a larger spatial scale, we additionally collected 85 specimens of both species from 27 locations across Germany (*N* = 21), Austria (*N* = 1), Denmark (*N* = 1), France (*N* = 3), and Scotland (*N* = 1) (Table S1).

In May 2011, before start of flowering, a total of 271 *Juncus* individuals not a priori assigned to either *J*. *effusus* or *J. conglomeratus* were haphazardly selected at the main study site in an area of 100 m × 600 m. For each individual, a single stem was marked and observed every other day until start of flowering, and the date of first flowering was recorded. In the field, the following morphological traits were measured for these stems: absolute stem height, absolute length of the lower bract (spathe length) and relative to stem height (spathe: stem), absolute length of the upper cataphyll (cataphyll) and relative to the stem height (cataphyll: stem), inflorescence volume computed using length (*l*), width (*w*) and depth (*d*) of the inflorescence and assuming an ellipsoid shape (V = 4/3*πlwd*), and flower density as the ratio of the number of flowers in the inflorescence and its volume. These quantitative traits are relatively easy to assess in the field and additionally have been frequently used for diagnostic purposes in this species complex. Furthermore, after fruit ripening, stem and inflorescence were harvested for laboratory analyses. First, the number of stem ridges 2–3 cm below the inflorescence was counted using a binocular, and second, seed length and width were measured by optical scanning with high resolution and applying image analysis implemented in WinSeedle (Regent Instruments Inc., Québec, Canada). Dried material from each stem was used for molecular analyses.

Chromosome counts were obtained from fresh root tips of plants raised in the greenhouse. Pretreatment and maceration followed Schwarzacher et al. (1980). Squash preparations were DAPI stained with Vectashield medium (Axxora, Lörrach, Gemany) and screened for metaphase chromosome spreads using an Axioskop2 plus (Zeiss, Jena, Germany), which were photographed for later chromosome counting.

In *Juncus* spp., herbivory by the moth genus *Coleophora* feeding on seeds has been reported frequently (Hård av Segerstad 1940; Randall 1986; Ellison 1991). Thus, predispersal seed predation may contribute to ecological differentiation between sympatric lineages. Hence, for each inflorescence, the proportion of capsules damaged by herbivore activity was recorded as an estimate for putative selective pressure by predispersal seed predation. Soil moisture content for each individual location was measured to assess potential small-scale habitat differentiation: After 2 weeks without rain, a sample of the upper 15 cm soil was taken as close as possible to each individual and soil moisture content was estimated by comparing fresh and dry weight.

To explicitly quantitate overlap in flowering time between the two *J*. *effusus* lineages at the study site, in June 2014, at the start of flowering, a total of 40 individuals were marked and all stems with open flowers were censused two or three times a week until the end of flowering.

### Molecular analyses

DNA extraction, amplification, and genotyping for loci AY493568, Jeff04, Jeff10, Jeff11, Jeff29, Jeff36, and five newly developed microsatellite loci (Table S2) followed Michalski and Durka (2012). In total, genotypic information was obtained for 356 individuals from 11 nuclear microsatellite loci. Maternally inherited chloroplast haplotypes were obtained by sequencing the intergenic spacer *rps*12-*clp*P (see Supporting information for detailed methods).

### Genetic data analysis

For the microsatellite data set, we used principal coordinate analysis (PCoA) of genotypic pairwise distances to visualize the similarity among individuals as implemented in GenAlEx v6.5 (Peakall and Smouse 2012). To test for genetic structuring, we used the Bayesian clustering approach implemented in STRUCTURE v.2.3.3 (Pritchard et al. 2000; Falush et al. 2003, 2007) (see Supporting information for detailed methods). Accounting for the fact that self-fertilization and inbreeding are very likely in the lineages studied, we also used InStruct (Gao et al. 2007) as an alternative to STRUCTURE which includes selfing in the respective model. Genetic diversity within gene diversity *H*_*E*_, and allelic richness corrected for sample size (*A*_*R*_) and genetic differentiation (*F*_*ST*_) among lineages (without hybrids, see below) were assessed using FSTAT v. 2.9.3 (Goudet 1995). Chloroplast sequences were aligned in Geneious Pro 5.6.5 (Biomatters 2012) using the MUSCLE algorithm (Edgar 2004) and default options. A haplotype network was created using statistical parsimony as implemented in TCS 1.21 (Clement et al. 2000). The connection limit between haplotypes was fixed to seven steps.

### Hybrid detection

Based on the genotypic data, hybrid status to each sample was assigned using NewHybrids 1.1 beta (Anderson and Thompson 2002). The program uses Bayesian inference to compute posterior probabilities for each sample to belong to genotype frequency classes. Here, posterior probabilities were calculated for six different classes that can arise after two generations of crossing between two parental populations (Anderson and Thompson 2002): Pure individuals of either population, F1 hybrids, second-generation hybrids (F2), and backcrosses between F1 hybrids with either parent (see Supporting information for detailed methods).

### Morphometric, ecological, and karyological analyses

For all pure individuals, we tested for differences in morphological traits, date of first flowering, and soil moisture content among groups identified with STRUCTURE by one-way ANOVA. The degree of herbivory was compared using a generalized linear model with quasibinomial errors to account for overdispersion. Multiple comparisons were carried out using post hoc Tukey tests. A principal component analysis (PCA) as well as a linear discriminant analysis on all standardized traits and individuals was performed to assess the morphological differentiation among genetically defined groups. Diagnostic traits were identified by correlating logit-transformed posteriors of the discriminant analysis against individual traits. For all analyses, inflorescence volume and flower density were log-transformed to ensure approximate variance homogeneity and normal distribution of residuals. Morphometric analyses were performed in the R environment v. 2.15.1 (R Core Team 2012) and using the package “MASS” (Venables and Ripley 2002).

Flowering overlap within and between *J*. *effusus* lineages, with individuals genetically assigned to either group as described above, was first quantified as among-individual synchrony (mean *r*), calculated as mean of all Pearson correlation coefficients of the proportion of flowering stems per individual and census across all pairwise individual comparisons (Koenig et al. 2003; Michalski and Durka 2007b). Synchrony was first calculated within lineages, second between groups only considering correlations between pairs from different lineages, and third treating all individuals as one group. Differences in synchrony were assumed to be significant if the 95% confidence interval derived from bootstrapping individuals 1000 times did not overlap. Second, overlap was quantified as lineage-specific probability of hybrid formation by the following approach (cf. Martin and Willis 2007): Assuming random mating, for each census *n* and lineage *i*, the probability of mating events with the other lineage *j* was calculated as 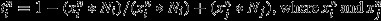 are the mean proportions of flowering inflorescences per individual and lineage; *N*_*i*_ and *N*_*j*_ are the absolute number of individuals per lineage censused in 2011, assuming that these numbers reflect actual individual frequencies in the population. The probability of hybrid formation across the whole flowering period (*P*_*i*_) was then calculated as the mean proportion of flowering inflorescences per census *x*_*i*_ relative to the cumulated proportion across all censuses, multiplied by the probability of mating events with the other lineage for that day. Eventually, this quantity was summed up across all censuses *n*:

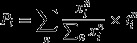


A 95% confidence interval for the estimates was derived from bootstrapping individuals within lineages 1000 times.

Chromosome counts were analyzed using linear mixed-effect models with genetically defined groups as fixed and individual as random effect using “lme4” for R (Bates et al. 2013). Significance of the group effect was tested by comparing models with and without groups as fixed effect.

### Hybrid fitness

To test whether first- and second-generation hybrids suffer from reduced fitness due to lower seed quality, we compared seed weight, approximated by seed volume, and germination percentage between selected hybrid samples (posterior probability to be either a F1 or F2 hybrid >0.95; *N* = 7) and arbitrarily selected pure individuals of all parental groups (*N* = 6–10). For the germination trial, between 15 and 126 seeds (mean # of seeds = 63) per individual were sown in plastic Petri dishes on filter paper soaked with tap water. Dishes were placed in a climatic chamber with a cycle of 18 h of light at 25°C followed by 6 h of darkness at 15°C known to provide optimal germination conditions (S. G. Michalski, unpubl. data). Germination success was assessed four times with a 7-day interval between counts. We tested for significant differences in seed volume between groups by one-way ANOVA. Germination success was compared using a generalized linear model with quasibinomial errors.

## Results

### Molecular analyses

**Figure 1:**
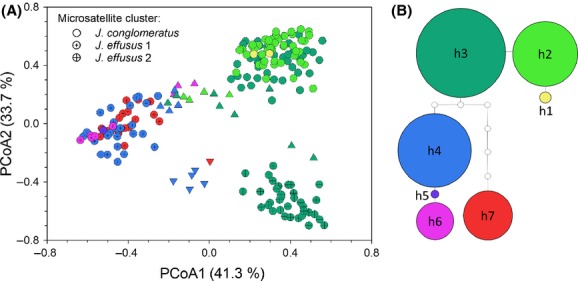
Diversity at nuclear microsatellite and chloroplast sequence level including samples from the main study site, across Germany and other countries. (A) PCoA of microsatellite genotypic distances. Circles represent pure individuals belonging to the microsatellite clusters detected by STRUCTURE: *Juncus conglomeratus* (upper right), *J*. *effusus* 1 (left, small cross), and *J*. *effusus* 2 (lower right, large cross). Triangles represent hybrids identified with NewHybrids. Fill color corresponds to chloroplast haplotypes. (B) Most parsimonious chloroplast haplotype network. Area of circles is proportional to the frequency of haplotypes in the data set. Haplotype color corresponds to the fill color of symbols in A.

In a PCoA of the microsatellite data for 271 individuals from the study site and 85 individuals from other European sites, 75% of the total genetic variation could be explained by the first two axes (Fig.1A). Samples formed a triangular pattern formed by three larger groups, two of which corresponded to *Juncus effusus,* which formed two groups (*eff1*, *eff2*), separated mainly along the first axis. Genotypes putatively of *J*. *conglomeratus* (*cong*) grouped together well separated from *eff1* along the first and from *eff2* along the second axis. This pattern was evident for genotypes from the main study site as well as for the additional samples (Fig. S1). Similar to the PCoA, the Bayesian cluster analysis with STRUCTURE clearly indicated the presence of three clusters (*K *=* *3, Δ*K *= 8703, mean S = 0.999, Fig. S2), reflecting the PCoA groups. The analysis with InStruct led to the same conclusion (data not shown). More than 80% of samples were unambiguously assigned to one of the clusters (mean individual posterior probability >0.95).

Gene diversity (*H*_*E*_) and allelic richness (*A*_*R*_) were similar for pure *J*. *conglomeratus* (*H*_*E*_ = 0.47, *A*_*R*_ = 4.1) and pooled *J*. *effusus* samples (*H*_*E*_ = 0.48, *A*_*R*_ = 3.6). However, treated separately, both *J*. *effusus* groups had lower genetic diversity values compared to *J*. *conglomeratus* (*eff1*: *H*_*E*_ = 0.21, *A*_*R*_ = 2.7; *eff2*: *H*_*E*_ = 0.24, *A*_*R*_ = 2.4, Table S3). Microsatellite differentiation among groups was substantial (*F*_*ST*_ = 0.57, 0.57, and 0.72, for comparisons *cong*-*eff1*, *cong*-*eff2,* and *eff1*-*eff2*, respectively).

Based on the NewHybrids results, about seven percent of all samples (*N* = 25) were assigned to be of hybrid origin (Fig. S3). Hybrids were detected between *conglomeratus* and *eff1* (*N* = 17, 68%), between *eff1 and eff2* (*N* = 6, 24%), and between *conglomeratus* and *eff2* (*N* = 2, 8%). Only seven hybrids (between *J*. *conglomeratus* and *eff1*) were likely to be F1 hybrids (posterior probability >0.9), whereas all others were later generation hybrids (F2, *N* = 9) or without clear assignment to either genotype frequency class.

Sequencing the intergenic spacer *rps*12-*clp*P revealed seven chloroplast haplotypes (GenBank accessions h1–h7: KF420421–KF420427, Fig.1B). Haplotypes h1–h2 were found in *J*. *conglomeratus* only; h3 was shared between *J*. *conglomeratus* and *eff2*; whereas haplotypes h4–h7 were detected only in *eff1*. While haplotypes found in *eff2* and *cong* differed by only 1 or 2 steps, *eff1* haplotypes differed from *eff2* and *cong* by 3–6 steps.

Putative hybrids between *conglomeratus* and *eff1* exhibited haplotypes of both parental species: h2 (*N* = 4, 25%), h3 (*N* = 5, 31%), h4 (*N* = 4, 25%), and h6 (*N* = 3, 19%). The two hybrids between *cong* and *eff2* shared haplotype h3. All hybrids between both *J*. *effusus* groups exhibited haplotypes of *eff1*: h4 (*N* = 5, 83%) and h7 which appeared only once (17%).

### Morphometric and karyological analyses

**Figure 2:**
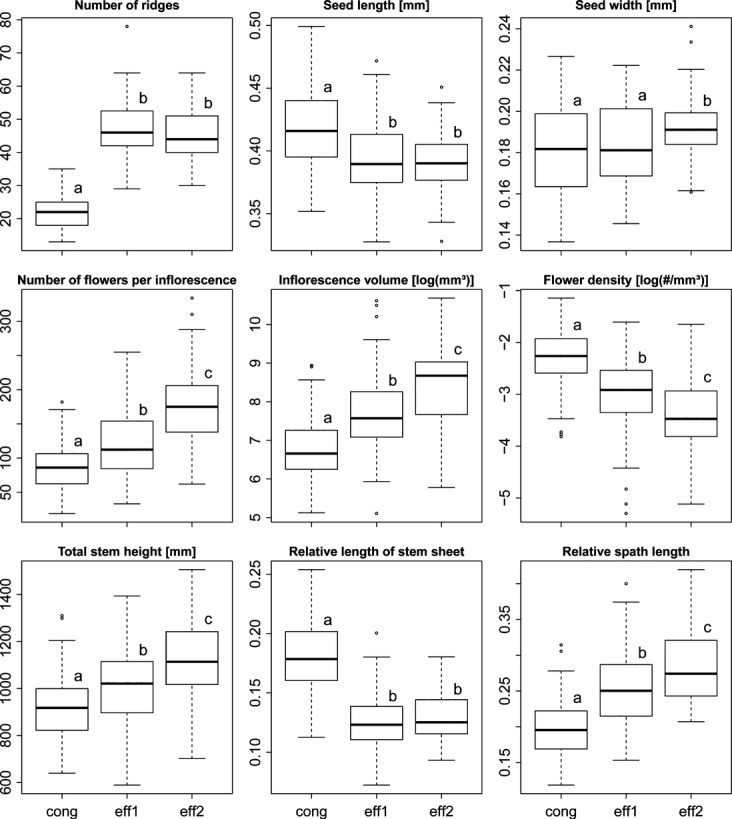
Differences in nine measured morphological traits between genetically defined groups: *Juncus conglomeratus* (cong), *J*. *effusus* 1 (eff1), and *J*. *effusus* 2 (eff2). Putative hybrids between groups were not considered. Different letters above boxes indicate significant differences between groups.

**Figure 3:**
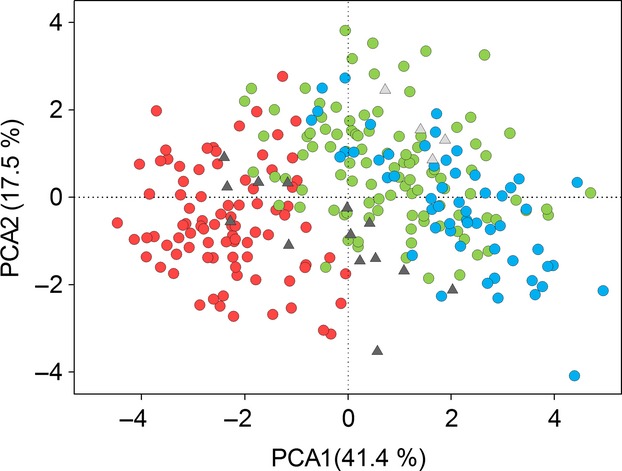
Studied individuals plotted in the trait space spanned by the first two axes of a principal component analysis on all measured morphological traits. Genetically defined pure groups are colored: *Juncus conglomeratus* (cong, red), *J*. *effusus* 1 (eff1, green), and *J*. *effusus* 2 (eff2, blue). Triangles represent genetically identified hybrids (dark gray *J*. *conglomeratus* x *J*. *effusus*, light gray *J*. *effusus eff1* × *eff2*).

For the three genetically identified groups (*cong*, *eff1*, and *eff2*), ANOVA found significant differences among groups for all morphological traits measured (*F *>* *5.78, *P *<* *0.01). Pairwise comparisons between pure groups showed that *cong* consistently differed from both *eff1* and *eff2* in all measured traits except for seed width. Furthermore, *eff1* differed significantly from *eff2* in eight of 12 traits assessed (Fig.2). However, *eff1* showed often intermediate values between or similar values to either *eff2* or *cong*. Only absolute cataphyll length was on average lower in *eff1* than in the two other groups (*P *<* *0.03). Consequently, in a PCA of all morphological traits with the first two axes explaining 58.9% of the total variation, *J*. *conglomeratus* separated relatively well from both *eff1* and *eff2* which overlapped substantially (Fig.3). Corroborating these results, linear discriminant analysis was able to assign 99% of pure individuals correctly to *J*. c*onglomeratus* but only 86% and 59% of individuals to groups *eff1* and *eff2*, respectively. *Juncus conglomeratus* was best separated from both *J*. *effusus* groups by the number of ridges and relative cataphyll length (*r* > 0.78, *P* < 0.001), and *eff1* and *eff2* were best distinguished by spathe length and the number of flowers per inflorescence (*r* > 0.64, *P* < 0.001). Chromosome counts were obtained for 12 individuals (Table S1: *N* = 2, 4, and 6 for *cong*, *eff1* and *eff2*, respectively) from six to 38 chromosome spreads per individual (mean 20.8). Clean spreads were difficult to obtain; hence, counts varied between 38 and 44, but did not differ significantly among groups (mean 2n ± SE = 40.94 ± 1.19, ANOVA *P* > 0.94).

### Site conditions, phenology, and herbivory

**Figure 4:**
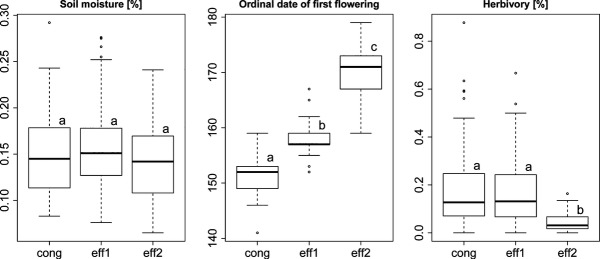
Ecological differences among genetically defined lineages: *Juncus conglomeratus* (cong), *J*. *effusus* 1 (eff1), and *J*. *effusus* 2 (eff2). Putative hybrids between groups were not considered. Different letters above boxes indicate significant differences between groups.

Soil moisture content did not differ among the three groups. Also, no obvious spatial clustering was evident, with individuals from all groups scattered across the main study site (Fig. S4). Groups differed significantly in the mean date of first flowering with *J*. *conglomeratus* blooming first, followed 7 days later by *eff1* and another 12 days later by *eff2* (Fig.4). Similarly, in 2014, peak flowering of *eff2* was 10–14 days later than that of *eff1*. Flowering lasted more than 20 days for *eff1* but only approximately 17–20 days for *eff2* with some overlap between the lineages (Fig. S5). Among-individual synchrony within the *J*. *effusus* groups was high (mean *r* = 0.43, 95% CI: 0.31–0.62 and mean *r* = 0.80 (0.73–0.90) for *eff1* and *eff2*, respectively) and significantly different from overall synchrony (mean *r* = 0.17 (0.14–0.27)). A significant asynchrony was found in the comparison between both groups (mean *r* = −0.22 (−0.34 to −0.07). Flowering overlap measured as linage specific probability of hybrid formation under the assumption of random mating was *P*_eff1_ = 0.08 (0.06–0.11) and *P*_eff2_* *= 0.07 (0.05–0.09).

The degree of herbivory on seeds measured as percentage of damaged capsules ranged from 0 to 88%, with the earlier flowering *cong* and *eff1* being similarly (*P* = 0.495), but significantly more heavily affected than the later flowering *eff2* (*P* < 0.001; 19; 17 and 4%, respectively; Fig.4, Fig. S6). Hybrids between *eff1* and *eff2* showed phenological values intermediate between the parental groups as well as intermediate levels of herbivory (mean ordinal date of first flowering = 158.4, 170.0, and 163.2; mean percentage of damaged capsules per inflorescence = 17.2, 4.4, and 10.2, for *eff1*, *eff2,* and hybrids, respectively).

### Hybrid seed quality

Hybrids pooled into one group did not differ in seed volume from pure lineages (*P* = 0.62). This result did not change when hybrids were separated in two groups (*J*. *conglomeratus* × *J*. *effusus* and *eff1 *×* eff2, P* = 0.50). All hybrids produced viable seeds (>30% individual germination percentage). Mean germination percentage after 4 weeks was >70% in all groups. In neither week, germination success differed significantly among groups. This result did not change when hybrids were separated in two groups.

## Discussion

### Genetic differentiation

Our results show that *Juncus effusus* in Europe is composed of two genetically well-differentiated lineages (*eff1* and *eff2*). In higher plants, cytotypes might constitute a significant component of cryptic diversity (e.g., Halverson et al. 2008; Koch et al. 2013; Muenzbergova et al. 2013). For example, in *Juncus biglumis* L., two genetically separated and occasionally co-occurring linages were found to differ in their ploidy levels (Schönswetter et al. 2007). However, karyotyping individuals representing both lineages of *J*. *effusus* and *J*. *conglomeratus* did not reveal significant differences suggesting a chromosome number of 2n = 42 common for all groups.

The two linages within *J*. *effusus* differed not only at nuclear marker level, but also showed different chloroplast haplotypes. At the nuclear marker level, we found lineages *eff1* and *eff2* to show similar levels of diversity. At the chloroplast level, however, *eff1* harbored four distinct haplotypes contrasting to *eff2* with only one haplotype which is, moreover, shared with *J*. *conglomeratus*. Such haplotype sharing between species has been reported from numerous taxa (Petit et al. 1997; Gutiérrez Larena et al. 2002; Gardner et al. 2004; Jakob and Blattner 2006). Horizontal gene transfer via hybridization as well as incomplete lineage sorting may account for such patterns (Maddison 1997).

Surprisingly, the genetic variation detected in samples across Europe was almost completely represented in the samples from the main study site (Fig. S1). Although wind-pollinated, extensive gene flow by pollen is unlikely to result in the observed lack of geographic genetic structuring because inbreeding coefficients for individual lineages were large (*F*_*IS*_ > 0.85) indicating high selfing rates, supporting earlier results (Michalski and Durka 2010, 2012). Selfing does not necessarily result in low within-population genetic diversity (Chauvet et al. 2004; Abbott et al. 2007; Michalski and Durka 2007a; Leger et al. 2009). Instead, high seed production and high seed dispersal potential mediated by wind, water, and animals (Richards and Clapham 1941b), and long-term persistent seed banks (Thompson et al. 1997) may account for the low spatial genetic structuring found.

### Morphological differentiation

The three genetic groups were found to differ significantly in a number of morphological traits (Fig.2). In line with other studies, the number of stem ridges discriminated best between *J*. *effusus* and *J*. *conglomeratus* (e.g., Stabbetorp 1989). Also relative cataphyll length provided a relatively good diagnostic certainty in the field (O'Mahony 2002). All other traits, despite significant differences in the mean values, showed a substantial overlap among lineages. In particular, *eff1* often showed intermediate values between *J*. *conglomeratus* and *eff2*, for example, for inflorescence traits like flower density. Thus, a distinction between the two lineages in *J*. *effusus* based on the assessed morphological traits was not possible due to the large overlap of trait values, justifying them as “cryptic” lineages. In *J*. *effusus,* it has been shown that flower density, and hence the compactness of the inflorescence, is to some extent genetically controlled in contrast to spathe length (Agnew 1968). Interestingly, some authors distinguished a type of *J*. *effusus* with compact inflorescences, similar to that of *J*. *conglomeratus* (*J*. *effusus* var. *subglomeratus* DC or var. *compactus* Lej. & Courtois) (Buchenau 1890; Fernald and Wiegand 1910; Tweed and Woodhead 1946; Fernández-Carvajal 1982), possibly corresponding to the genetically defined lineage *eff1* within *J*. *effusus*.

### Ecological differentiation

Ecological differentiation due to abiotic conditions, phenology, or biotic interactions may contribute to lineage divergence (Givnish 2010). In wetland ecosystems, soil moisture content describes an important environmental parameter and in our main study population indeed reflected a substantial gradient (range of soil moisture content: 7%–29%). However, although *J*. *conglomeratus* has been described as more tolerant to drier soil conditions than *J*. *effusus* (Strelkova 1928; Richards and Clapham 1941a), they did not differ in local soil moisture content, neither did the two *J*. *effusus* lineages. Still, we cannot rule out resource partitioning for other soil parameters such as soil pH or nutrient availability.

The impact of premating barriers and local individual density distributions on reproductive isolation is well known from a number of studies (e.g., Lepais et al. 2009; Field et al. 2011). Here, we demonstrated a strong differentiation in flowering time among the lineages with *J*. *conglomeratus* flowering first, followed by *eff1* and much later by *eff2* (Fig.4, Fig. S5).

Assuming a flowering duration of 14–20 days for *J*. *conglomeratus* (cf. Michalski and Durka 2007b), our results suggest that the overlap in flowering between *eff2* and *J*. *conglomeratus* is almost nonexisting, which is consequently reflected in the lowest number of putative hybrids detected between these lineages (*N* = 2). Flowering time is less probable to provide a reproductive barrier for the temporally intermediate *eff1*, which indeed contributed most to hybrid formation. Although both *J*. *effusus* lineages showed some degree of overlap in flowering time, the probability of hybrid formation was <10% even under the unlikely assumption of random mating. As outcrossing seems the exception rather than the rule in this species group, flowering time differentiation successfully contributes to reproductive isolation between the lineages.

Predispersal seed predation by *Coleophora* spp. may strongly affect seed production in *Juncus* (Hård av Segerstad 1940; Randall 1986). Thus, in addition to providing a reproductive barrier, flowering time may be under strong disruptive selection pressure. Although the two early flowering lineages *cong* and *eff1* suffered on average similarly strong from seed predation, both the earliest flowering individuals of *cong* and the later flowering *eff2* were less affected (Fig. S6).

### Hybridization and hybrid fitness

Incomplete isolation or the breakdown of reproductive barriers can lead to hybridization which in turn may counteract lineage formation (Garcia et al. 2011). Indeed, we found evidence for hybridization between all three studied genetic lineages, mostly between *eff1* and *J*. *conglomeratus*.

Assuming that the number of hybrids in our sample is representative for the natural hybridization frequency, rates are relatively high with 7% for the study site. There was no evidence for cytoplasmic introgression into either group. However*, eff1* × *eff2* hybrids were biased toward chloroplast haplotypes of *eff1* (Fig.1), suggesting a stronger maternal contribution of *eff1* than *eff2* to hybrid formation. As flowers are wind pollinated and homogamous and other unidirectional acting barriers are not obvious, such asymmetric pollen flow must be caused by differences in local abundance of parental lineages (Lepais et al. 2009). In fact, at the two sites covered by ≥30 samples, *eff1* was more common than *eff2* (Table S1).

All investigated putative hybrids produced seeds of similar quality and had similar germination rates compared to the parental lineages. On the other hand, hybrids between the *J*. *effusus* lineages showed a higher predispersal seed loss than the later flowering parental lineage suggesting that fitness might be negatively affected by hybridization. The potential risk of extinction of these sympatric lineages may depend on whether other additional components of fitness are significantly decreased in hybrids. Maintenance of sympatric lineages might be fostered by life-history traits such as a long generation time and/or a selfing mating system (Wolf et al. 2001).

### Evolutionary origin of cryptic lineages in *J*. *effusus*

Sympatric coexisting and morphologically similar but genetically differentiated lineages can be explained evolutionary by either lineage differentiation in sympatry or by a secondary contact between lineages formed in allopatry. Our results on genetic and ecological differentiation patterns for the two *J*. *effusus* lineages can be interpreted in favor of the secondary-contact hypothesis. First, only little evidence could be found for a clear ecological and functional separation between the lineages except for a pronounced differentiation in flowering time and correlated herbivore pressure. Differences in flowering phenologies among closely related sympatric plant species have been reported frequently (McIntosh 2002; Martin and Willis 2007; Ferriol et al. 2009; Pascarella 2011). Although disruptive selection imposed by seed predation may contribute to flowering time differentiation and reproductive isolation (Elzinga et al. 2007), it not necessarily needs to be invoked to explain sympatric differentiation. Devaux and Lande (2008) modeled the evolution of flowering time using a multigenic model with assortative mating and mutation showing that sympatric formation of allochronic-isolated lineages is favored even without selection under conditions that are partly met in our study species, such as low inbreeding depression (Edgar 1964) or the absence of pollen limitation (Michalski and Durka 2007b, 2010). However, differentiation in flowering time between lineages may similarly arise in allopatry because of stochastic and/or selective divergence. Indeed, the degree of quantitative differentiation for the start of flowering (*P*_*ST*_ computed with *c *= 1 and *h*^2^ = 0.5, sensu Brommer 2011) between the two *J*. *effusus* lineages is comparable with that of differentiation at nuclear marker level (*P*_*ST*_ = 0.79 vs. *F*_*ST*_ = 0.72), suggesting that a simple stochastic mode of lineage divergence alone can explain the observed pattern. Second, the absolute magnitude of this neutral genetic differentiation between the *J*. *effusus* lineages, despite a lack of postzygotic reproductive isolation, is unlikely to be the result of a speciation process and a prolonged coexistence in sympatry.

In conclusion, while *J*. *effusus* and *J*. *conglomeratus* form morphologically distinct groups, they in fact represent a system of three genetically differentiated, homoploid lineages with *J*. *effusus* showing evidence for a deep genomic split between two morphologically cryptic groups suggesting a complex evolutionary history within the species. While not completely preventing hybridization among sympatric lineages, differences in flowering time and a selfing mating system are likely to promote reproductive isolation. *Juncus effusus* is a model plant for research on wetland ecosystem functioning and remediation approaches and studied with respect to pest control or secondary metabolites. Hence, future work on that species will need to include and address the impact of the distinct within-species diversity described here.

## Data Accessibility

The raw data underlying the main results of the presented study has been deposited at the dryad repository (doi:10.5061/dryad.bk5hk).

## Supporting Information

**Methods S1.** Detailed methods for genetic marker analysis and hybrid detection. **Table S1.** Information on samples and locations included in the genetic analyses. **Table S2.** Information on newly developed microsatellite markers for *Juncus effusus*. **Table S3.** Summary statistics for microsatellite loci. **Figure S1.** Comparison of diversity patterns at microsatellite and chloroplast sequence level for samples from the main study site and other locations. **Figure S2.** STRUCTURE summary statistics. **Figure S3.** Posterior probabilities for different genotypic frequency classes for hybrid individuals. **Figure S4.** Spatial distribution of samples at the main study site. **Figure S5.** Proportion of flowering inflorescences against time. **Figure S6.** Proportion of damaged capsules against date of first flowering.

## References

[b1] Abbott RJ, Ireland HE, Rogers HJ (2007). Population decline despite high genetic diversity in the new allopolyploid species *Senecio cambrensis* (Asteraceae). Mol. Ecol.

[b2] Agnew ADQ (1961). The ecology of *Juncus effusus* L. in North Wales. J. Ecol.

[b3] Agnew ADQ (1968). The interspecific relationship of *Juncus effusus* and *J**conglomeratus* in Britain. Watsonia.

[b4] Anderson EC, Thompson EA (2002). A model-based method for identifying species hybrids using multilocus genetic data. Genetics.

[b5] Bates D, Maechler M, Bolker B, Walker S (2013). lme4: linear mixed-effect models using Eigen and S4. R package version 1.0-4.

[b6] Bickford D, Lohman DJ, Sodhi NS, Ng PKL, Meier R, Winker K (2007). Cryptic species as a window on diversity and conservation. Trends Ecol. Evol.

[b7] Biomatters (2012). Geneious Pro 5.6.5.

[b8] Borsch T, Limarino TO, Nee MH (2011). Phylogenetics of the neotropical liana genus *Pedersenia* (Amaranthaceae: Gomphrenoideae) and discovery of a new species from Bolivia based on molecules and morphology. Willdenowia.

[b9] Bower CC, Brown GR (2009). Pollinator specificity, cryptic species and geographical patterns in pollinator responses to sexually deceptive orchids in the genus *Chiloglottis*: the *Chiloglottis gunnii* complex. Aust. J. Bot.

[b10] Brommer JE (2011). Whither Pst? The approximation of Qst by Pst in evolutionary and conservation biology. J. Evol. Biol.

[b11] Buchenau F (1890). Monographia Juncacearum. Bot. Jahrb. Syst.

[b12] Buchenau F (1892). Ueber die Bestäubungs-Verhältnisse bei den Juncaceen. Jahrb. Wiss. Bot.

[b13] Chauvet S, Van der Velde M, Imbert E, Guillemin ML, Mayol M, Riba M (2004). Past and current gene flow in the selfing, wind-dispersed species *Mycelis muralis* in western Europe. Mol. Ecol.

[b14] Chesson P (2000). Mechanisms of maintanance of species diversity. Annu. Rev. Ecol. Syst.

[b15] Clement M, Posada D, Crandall KA (2000). TCS: a computer program to estimate gene genealogies. Mol. Ecol.

[b16] Devaux C, Lande R (2008). Incipient allochronic speciation due to non-selective assortative mating by flowering time, mutation and genetic drift. Proc. R. Soc. Lond. B Biol. Sci.

[b17] Drábková LZ (2013). A Survey of karyological phenomena in the Juncaceae with emphasis on chromosome number variation and evolution. Bot. Rev.

[b18] Edgar E (1964). The leafless species of *Juncus* in New Zealand. N. Z. J. Bot.

[b19] Edgar RC (2004). MUSCLE: multiple sequence alignment with high accuracy and high throughput. Nucleic Acids Res.

[b20] Ellis AG, Weis AE, Gaut BS (2006). Evolutionary radiation of “stone plants” in the genus *Argyroderma* (Aizoaceae): unraveling the effects of landscape, habitat, and flowering. Evolution.

[b21] Ellison AM (1991). Ecology of case-bearing moths (Lepidoptera, Coleophoridae) in a New-England salt-marsh. Environ. Entomol.

[b22] Elzinga JA, Atlan A, Biere A, Gigord L, Weis AE, Bernasconi G (2007). Time after time: flowering phenology and biotic interactions. Trends Ecol. Evol.

[b23] Ervin GN, Wetzel RG (2001). Seed fall and field germination of needlerush, *Juncus effusus* L. Aquat. Bot.

[b24] Esselstyn JA, Evans BJ, Sedlock JL, Khan FAA, Heaney LR (2012). Single-locus species delimitation: a test of the mixed Yule-coalescent model, with an empirical application to Philippine round-leaf bats. Proc. R. Soc. Lond. B Biol. Sci.

[b25] Falush D, Stephens M, Pritchard JK (2003). Inference of population structure using multilocus genotype data: linked loci and correlated allele frequencies. Genetics.

[b26] Falush D, Stephens M, Pritchard JK (2007). Inference of population structure using multilocus genotype data: dominant markers and null alleles. Mol. Ecol. Notes.

[b27] Fernald ML, Wiegand KM (1910). The North American variations of *Juncus effusus*. Rhodora.

[b28] Fernández-Carvajal MC (1982). Revisión del género *Juncus* L. en la Península Ibérica. II Subgéneros *Juncus* y *Genuini* Buchenau. An. Jard. Bot. Madr.

[b29] Ferriol M, Llorens L, Gil L, Boira H (2009). Influence of phenological barriers and habitat differentiation on the population genetic structure of the balearic endemic *Rhamnus ludovici-salvatoris* Chodat and *R**alaternus* L. Plant Syst. Evol.

[b30] Field DL, Ayre DJ, Whelan RJ, Young AG (2011). The importance of pre-mating barriers and the local demographic context for contemporary mating patterns in hybrid zones of *Eucalyptus aggregata* and *Eucalyptus rubida*. Mol. Ecol.

[b31] Fishman L, Aagaard J, Tuthill JC (2008). Toward the evolutionary genomics of gametophytic divergence: patterns of transmission ratio distortion in monkeyflower (*Mimulus*) hybrids reveal a complex genetic basis for conspecific pollen precedence. Evolution.

[b32] Flaxman SM, Wacholder AC, Feder JL, Nosil P (2014). Theoretical models of the influence of genomic architecture on the dynamics of speciation. Mol. Ecol.

[b33] Gao H, Williamson S, Bustamante CD (2007). A Markov Chain Monte Carlo approach for joint inference of population structure and inbreeding rates from multilocus genotype data. Genetics.

[b34] Garcia MG, Silva RS, Carniello MA, Veldman JW, Rossi AAB, de Oliveira LO (2011). Molecular evidence of cryptic speciation, historical range expansion, and recent intraspecific hybridization in the Neotropical seasonal forest tree *Cedrela fissilis* (Meliaceae). Mol. Phylogenet. Evol.

[b35] Gardner RC, De Lange PJ, Keeling DJ, Bowala T, Brown HA, Wright SD (2004). A late Quaternary phylogeography for *Metrosideros* (Myrtaceae) in New Zealand inferred from chloroplast DNA haplotypes. Biol. J. Linn. Soc.

[b36] Givnish TJ (2010). Ecology of plant speciation. Taxon.

[b37] Goudet J (1995). FSTAT (version 1.2): a computer program to calculate F-statistics. J. Hered.

[b38] Govindarajulu R, Hughes CE, Bailey CD (2011). Phylogenetic and population genetic analyses of diploid *Leucaena* (Leguminosae; Mimosoideae) reveal cryptic species diversity and patterns of divergent allopatric speciation. Am. J. Bot.

[b39] Gruber H, Wiessner A, Kuschk P, Kaestner M, Appenroth KJ (2008). Physiological responses of *Juncus effusus* (rush) to chromium and relevance for wastewater treatment in constructed wetlands. Int. J. Phytoremediat.

[b40] Gutiérrez Larena B, Fuertes Aguilar J, Nieto Feliner G (2002). Glacial-induced altitudinal migrations in *Armeria* (Plumbaginaceae) inferred from patterns of chloroplast DNA haplotype sharing. Mol. Ecol.

[b41] Halverson K, Heard SB, Nason JD, Stireman JO (2008). Origins, distribution, and local co-occurrence of polyploid cytotypes in Solidago altissima (Asteraceae). Am. J. Bot.

[b42] Hämet-Ahti L (1980). The *Juncus effusus* aggregate in eastern North America. Ann. Bot. Fenn.

[b43] Hård av Segerstad F (1940). Zur Systematik und Biologie von *Juncus effusus* L. und *Juncus conglomeratus* L. Acta Horti. Gothob.

[b44] Hendry AP, Lohmann LG, Conti E, Cracraft J, Crandall KA, Faith DP (2010). Evolutionary biology in biodiversity science, conservation, and policy: a call to action. Evolution.

[b45] Henry CS (1994). Singing and cryptic speciation insects. Trends Ecol. Evol.

[b46] Howard DJ (1999). Conspecific sperm and pollen precedence and speciation. Annu. Rev. Ecol. Syst.

[b47] Hubbell SP (2005). Neutral theory in community ecology and the hypothesis of functional equivalence. Funct. Ecol.

[b48] Jakob SS, Blattner FR (2006). A chloroplast genealogy of *Hordeum* (Poaceae): Long-term persisting haplotypes, incomplete lineage sorting, regional extinction, and the consequences for phylogenetic inference. Mol. Biol. Evol.

[b49] Kirschner J, Balslev H, Brooks RE, Clemants SE, Ertter B, Hämet-Ahti L (2002). Juncaceae 3: Juncus subg. Agathryon.

[b50] Koch MA, Scheriau C, Betzin A, Hohmann N, Sharbel TF (2013). Evolution of cryptic gene pools in Hypericum perforatum: the influence of reproductive system and gene flow. Ann. Bot.

[b51] Koenig WD, Kelly D, Sork VL, Duncan RP, Elkinton JS, Peltonen MS (2003). Dissecting components of population-level variation in seed production and the evolution of masting behavior. Oikos.

[b52] Křísa B (1962). Relations of the ecologica-phenological observation to the taxonomy of the species *Juncus effusus* L. s.l. Preslia.

[b53] Leger EA, Espeland EK, Merrill KR, Meyer SE (2009). Genetic variation and local adaptation at a cheatgrass (*Bromus tectorum*) invasion edge in western Nevada. Mol. Ecol.

[b54] Leibold MA, McPeek MA (2006). Coexistence of the niche and neutral perspectives in community ecology. Ecology.

[b55] Lepais O, Petit RJ, Guichoux E, Lavabre JE, Alberto F, Kremer A (2009). Species relative abundance and direction of introgression in oaks. Mol. Ecol.

[b56] Levin DA (2012). The long wait for hybrid sterility in flowering plants. New Phytol.

[b57] Maddison WP (1997). Gene trees in species trees. Syst. Biol.

[b58] Martin NH, Willis JH (2007). Ecological divergence associated with mating system causes nearly complete reproductive isolation between sympatric *Mimulus* species. Evolution.

[b59] McCorry M, Renou F (2003).

[b60] McIntosh ME (2002). Flowering phenology and reproductive output in two sister species of *Ferocactus* (Cactaceae). Plant Ecol.

[b61] Michalski SG, Durka W (2007a). High selfing and high inbreeding depression in peripheral populations of *Juncus atratus*. Mol. Ecol.

[b62] Michalski SG, Durka W (2007b). Synchronous pulsed flowering: analysis of the flowering phenology in *Juncus* (Juncaceae). Ann. Bot.

[b63] Michalski S, Durka W (2010). Pollen and ovule production in wind-pollinated species with special reference to *Juncus*. Plant Syst. Evol.

[b64] Michalski SG, Durka W (2012). Identification and characterization of microsatellite loci in the rush *Juncus effusus* (Juncaceae). Am. J. Bot.

[b65] Muenzbergova Z, Surinova M, Castro S (2013). Absence of gene flow between diploids and hexaploids of *Aster amellus* at multiple spatial scales. Heredity.

[b66] Ni LH, Li Q, Kong LF, Huang SQ, Li LJ (2012). DNA barcoding and phylogeny in the family Mactridae (Bivalvia: Heterodonta): evidence for cryptic species. Biochem. Syst. Ecol.

[b67] O'Mahony T (2002). The comparative morphology of *Juncus conglomeratus* L. (Compact Rush), *J.effusus* L. (Soft-rush) and their interspecific hybrid, *J*. × *kernreichgeltii* Jansen & Wachter ex Reichg. Ir. Bot. News.

[b68] Otto SP, Whitton J (2000). Polyploid incidence and evolution. Annu. Rev. Genet.

[b69] Pascarella JB (2011). The relationship between soil environmental factors and flowering phenology in two sympatric Southeastern *Gelsemium* species-Does habitat specialization determine differences in flowering time?. Castanea.

[b70] Peakall R, Smouse PE (2012). GenAlEx 6.5: genetic analysis in Excel. Population genetic software for teaching and research—an update. Bioinformatics.

[b71] Petit RJ, Pineau E, Demesure B, Bacilieri R, Ducousso A, Kremer A (1997). Chloroplast DNA footprints of postglacial recolonization by oaks. Proc. Natl Acad. Sci. USA.

[b72] Pritchard JK, Stephens M, Donnelly P (2000). Inference of population structure using multilocus genotype data. Genetics.

[b73] R Core Team (2012). R: a language and environment for statistical computing.

[b74] Ragupathy S, Newmaster SG, Murugesan M, Balasubramaniam V (2009). DNA barcoding discriminates a new cryptic grass species revealed in an ethnobotany study by the hill tribes of the Western Ghats in southern India. Mol. Ecol. Resour.

[b75] Ramsey J, Bradshaw HD, Schemske DW (2003). Components of reproductive isolation between the monkeyflowers *Mimulus lewisii* and *M**cardinalis* (Phrymaceae). Evolution.

[b76] Randall MGM (1986). The predation of predispersed *Juncus squarrosus* seeds by *Coleophora alticolella* (Lepidoptera) larvae over a range of altitued in northern England. Oecologia.

[b77] Richards MB, Clapham AR (1941a). Biological flora of the British Isles - *Juncus conglomeratus* L. (*J**communis α conglomeratus* E. Mey.; *J**leersii* Marsson). J. Ecol.

[b78] Richards PW, Clapham AR (1941b). Biological flora of the British Isles - *Juncus effusus* L. (*Juncus communis β effusus* E. May). J. Ecol.

[b79] Rieseberg LH, Willis JH (2007). Plant speciation. Science.

[b80] Savolainen V, Anstett MC, Lexer C, Hutton I, Clarkson JJ, Norup MV (2006). Sympatric speciation in palms on an oceanic island. Nature.

[b81] Schönswetter P, Suda J, Popp M, Weiss-Schneeweiss H, Brochmann C (2007). Circumpolar phylogeography of Juncus biglumis (Juncaceae) inferred from AFLP fingerprints, cpDNA sequences, nuclear DNA content and chromosome numbers. Mol. Phylogenet. Evol.

[b82] Schwarzacher T, Ambros P, Schweizer D (1980). Application of Giemsa banding to orchid karyotype analysis. Plant Syst. Evol.

[b83] Silvertown J, Servaes C, Biss P, Macleod D (2005). Reinforcement of reproductive isolation between adjacent populations in the Park Grass Experiment. Heredity.

[b84] Stabbetorp OE (1989). A morphological analysis of *Juncus effusus* and *J**conglomeratus*. Blyttia.

[b85] Strelkova OS (1928). Notes on the species J*uncus effusus* L. and *J**conglomeratus* L. Trudy LOE.

[b86] Tellier F, Vega JMA, Broitman BR, Vasquez JA, Valero M, Faugeron S (2011). The importance of having two species instead of one in kelp management: the *Lessonia nigrescens* species complex. Cah. Biol. Mar.

[b87] Thompson K, Bakker J, Bekker R (1997). The soil seed banks of North West Europe: methodology, density and longevity.

[b88] Tweed RD, Woodhead N (1946). A consideration of *Juncus effusus* L. and *Juncus conglomeratus* L. J. Ecol.

[b89] Venables WN, Ripley BD (2002). Modern applied statistics with S.

[b90] Weimarck H (1946). Studies in Juncaceae - with special reference to the species in Ethiopia and the Cape. Sven. Bot. Tidskr.

[b91] Whittall JB, Hellquist CB, Schneider EL, Hodges SA (2004). Cryptic species in an endangered pondweed community (*Potamogeton*, Potamogetonaceae) revealed by AFLP markers. Am. J. Bot.

[b92] Wilcox M (2010). A novel approach to the determination and identification of *Juncus *× *diffusus* Hoppe and *J*. × *kern-reichgeltii* Jansen & Wacht. ex Reichg. Watsonia.

[b93] Wolf DE, Takebayashi N, Rieseberg LH (2001). Predicting the risk of extinction through hybridization. Conserv. Biol.

[b94] Yang JB, Wang YP, Moller M, Gao LM, Wu D (2012). Applying plant DNA barcodes to identify species of *Parnassia* (Parnassiaceae). Mol. Ecol. Resour.

[b95] Zika P (2003). The native subspecies of *Juncus effusus* (Juncaceae) in western North America. Brittonia.

