# Supplementary material for: Separation in flowering time contributes to the maintenance of sympatric cryptic plant lineages
Source: Ecol Evol. 2015 May 8;5(11):2172–84. doi: 10.1002/ece3.1481 (PMC4461419; doi:10.1002/ece3.1481)
Supplement: Supplementary file 1 [file ece30005-2172-sd1.docx]

## *Ecology and Evolution* Supporting Information

Article title: Separation in flowering time contributes to the maintenance of sympatric cryptic plant lineages

Authors: Stefan G. Michalski and Walter Durka

The following Supporting Information is available for this article:

**Methods S1**

(a) Molecular analyses

Maternally inherited chloroplast haplotypes for all samples were obtained by sequencing the intergenic spacer *rps*12-*clp*P (Scarcelli et al. 2011). To increase amplification success a new backward primer *clp*P_Rjunc (5’-TGTGGCTGATCGTTTTTCTG-3’) was used. Reaction mixes (20 µl) included 5-20 ng of sample DNA, 2.5 ng of forward and backward primer each, 200 µM dNTPs (Roth), 2 µl of 10xDreamTaq Buffer (Fermentas), 1 unit of DreamTaq DNA polymerase (Fermentas) and 12.8 µl water. The thermocycler protocol was 95 °C (3min) followed by 35 cycles of 95 °C (30s), 56 °C (45s) and 72 °C (60s) and a final step at 72 °C (10 min). The product was directly cycle sequenced with the BigDye Terminator v.3.1 Cycle Sequencing Kit (Applied Biosystems), using the forward primer described in Scarcelli et al. (2011). A 10 µl reaction contained 2.1 µl 5x Sequencing Buffer (Applied Biosystems), 2.5 pmol primer, 0.5 µl BigDye Terminator v.3.1 and 1 µl of a 1:5 dilution of the template. The cycling scheme was 95°C (3 min), followed by 30 cycles at 95°C (20 s), 50°C (15 s) and 60°C (4 min) and a final step at 60°C (10 min). Samples were run on an ABI 3130 genetic analyzer.

(d) Genetic data analysis

To test for genetic structuring we used the Bayesian clustering approach implemented in STRUCTURE v.2.3.3 (Pritchard et al. 2000; Falush et al. 2003; Falush et al. 2007) using an admixture model with correlated allelic frequencies without prior information on sampling identity or origin. The log probability of data was calculated for genetic clusters *K* = 1 to *K*= 10 with 15 independent runs for each *K*. For each run the burnin period was 100000 iterations and the log probability of data was calculated from additional 500000 iterations. The most likely number of genetic clusters (*K*) was estimated using the Δ*K* method (Evanno et al. 2005). Δ*K* and similarity coefficients (S) among pairs of runs at a given *K* (Nordborg et al. 2005; Ehrich et al. 2007) were calculated using an published R script (Ehrich et al. 2007).

(e) Hybrid detection

Based on the genotypic data, hybrid status was assigned using NewHybrids 1.1beta (Anderson and Thompson 2002). The program uses Bayesian inference to compute posterior probabilities for each sample to belong to genotype frequency classes. Here, posterior probabilities were calculated for six different classes that can arise after two generations of crossing between two parental populations (Anderson and Thompson 2002): Pure individuals of either population, F1 hybrids, second generation hybrids (F2) and backcrosses between F1 hybrids with either parent. For mixing proportions and allelic frequencies Jeffreys-type prior distributions were assumed. As STRUCTURE indicated a tripartition of samples as most likely result and NewHybrids can handle only two parental populations, three separate analyses were conducted, each for one pair of clusters. Individuals were assigned to a cluster pair if added mean individual posterior probabilities across the 15 STRUCTURE runs for K=3 for both clusters exceeded 0.9. Using this approach, all but one individual could be assigned unambiguously to one or two cluster pairs, hence, that individual was included in all three analyses. In all analyses with NewHybrids complete-data log likelihood reached stability after a few hundred iterations of the MCMC sampler. Running the sampler from different starting conditions did not change the results. Hence, a burn-in phase of 10000 iterations was considered to be sufficient and posterior probabilities were calculated from 50000 additional iterations. Individuals were conservatively assigned to be hybrids only if in none of the analyses the posterior probability for belonging to either one of the pure parental populations was higher than 0.1. Otherwise, it could not be excluded that individuals belonged to one of the pure parental populations.

**Table S1**. Samples included in the genetic analyses and their position in the three STRUCTURE clusters. The class ‘hyb’ indicates admixed individuals without clear assignment. The letters behind the number of samples in this class indicates the type of admixture: a – individuals show admixture between cluster ‘cong’ and ‘eff1 ‘, b – between ‘cong’ and ‘eff2’, and c – between ‘eff1’ and ‘eff2’. An asterisk marks individuals for which chromosome counts were obtained (note that for Halle only one individual per group was karyologically analyzed).

|  |  |  |  | # of samples in *STRUCTURE* cluster | | | |
| --- | --- | --- | --- | --- | --- | --- | --- |
| Location | *N* | Latitude [°] | Longitude [°] | cong | eff1 | eff2 | hyb |
| Halle, Germany | 271 | 51.511 | 11.927 | 88 | 104* | 59* | 20abc |
| Mandlinger Moor, Austria | 1 | 47.403 | 13.563 |  |  | 1* |  |
| Soedervig, Denmark | 1 | 56.132 | 8.134 | 1* |  |  |  |
| Beulotte-Saint-Laurent, France | 1 | 47.848 | 6.682 |  | 1* |  |  |
| Village de Bavella (Corsica), France | 1 | 41.816 | 9.207 | 1* |  |  |  |
| Campénéac, France | 1 | 47.959 | -2.239 |  | 1 |  |  |
| Brand-Erbisdorf, Germany | 1 | 50.850 | 13.319 |  |  |  | 1a |
| Großhartmannsdorf, Germany | 3 | 50.783 | 13.326 |  | 3 |  |  |
| Weisdin, Germany | 1 | 53.404 | 13.113 |  |  | 1* |  |
| Lüneburg, Germany | 1 | 53.225 | 10.417 |  | 1* |  |  |
| Selkemühle, Germany | 1 | 51.675 | 11.187 |  |  | 1 |  |
| Leipzig, Germany | 1 | 51.365 | 12.413 |  |  | 1* |  |
| Schorfheide, Germany | 1 | 52.990 | 13.604 |  | 1 |  |  |
| Neustrehlitz, Germany | 1 | 53.324 | 13.099 |  |  | 1 |  |
| Gelsenkirchen-Buer, Germany | 1 | 51.570 | 7.067 |  |  | 1* |  |
| Jemmeritzer Moor, Germany | 1 | 52.637 | 11.262 |  | 1 |  |  |
| Pestruper Felder, Germany | 1 | 52.875 | 8.451 |  | 1 |  |  |
| Botanical Garden Jena, Germany | 1 | 50.931 | 11.585 |  |  | 1* |  |
| Jävenitzer Moor, Germany | 1 | 52.503 | 11.472 |  | 1* |  |  |
| Schäferbachtal, Germany | 6 | 51.653 | 11.032 | 3 | 3 |  |  |
| Freiberg, Germany | 4 | 50.942 | 13.307 | 1 | 3 |  |  |
| Heiligendamm, Germany | 8 | 54.144 | 11.832 | 4 |  | 3 | 1c |
| Nienhagen, Germany | 4 | 54.162 | 11.937 | 2 |  | 2 |  |
| Erpholzheim, Germany | 3 | 49.491 | 8.242 | 3 |  |  |  |
| Hohes Holz, Germany | 6 | 52.089 | 11.226 | 3 |  | 3 |  |
| Lopausee, Germany | 30 | 53.129 | 10.224 | 8 | 19 | 2 | 1a |
| Harz, Bremer Teich, Germany | 3 | 51.686 | 11.110 |  |  | 3 |  |
| Dunfermline, Scotland | 1 | 56.060 | -3.387 |  |  |  | 1a |

**Table S2**. Five microsatellite loci for *Juncus effusus* newly developed based on data and methods of Michalski & Durka (2012).

| Locus | Accession number | Repeat motif | Size range | Primer sequence (5′ – 3′ ) | 5'-tag | Label |
| --- | --- | --- | --- | --- | --- | --- |
| Jeff058 | KF380880 | (AAG)_7_ | 213-227 | F: TTCTTCTCTTCGTTTCAAG | M13R | NED |
|  |  |  |  | R: ATTTGGCGTAGATATCAAAG | GTTT |  |
| Jeff059 | KF380881 | (AAG)_7_ | 113-124 | F: GTCGCAAATTCCTAATTAAC | GTTT | FAM |
|  |  |  |  | R: GAAGAACCCACTCCATTATC | M13R |  |
| Jeff067 | KF380882 | (AAT)_7_ | 240-249 | F: TGCAGTTTATCCGTGAGTAC | GTT | VIC |
|  |  |  |  | R: AAACAGAAGGATGAATTAGC | M13R |  |
| Jeff069 | KF380883 | (AATT)_7_ | 191-203 | F: TGGGTTTCGTTTGTATTTAC | CAG | PET |
|  |  |  |  | R: GTTTAGCCACTTCATGTTAGTTTC | GTTT |  |
| Jeff074 | KF380884 | (ACC)_7_ | 287-335 | F: GTTTCGTCTGGATCGTATTATCAG | GTTT | FAM |
|  |  |  |  | R: CAAATGCCTCTCTTTCATAG | M13R |  |

**Table S3**. Summary statistics, i.e. Nei's (1987) gene diversity and, in brackets, allelic richness for individual microsatellite loci in the three lineages studied (cong – *Juncus conglomeratus*; eff1 – *J*. *effusus* lineage 1; eff2 – *J*. *effusus* lineage 2).

|  | Lineage | | |
| --- | --- | --- | --- |
| Locus | *cong* | *eff1* | *eff2* |
| AY493568 | 0.336 (5) | 0.655 (7) | 0.05 (3) |
| Jeff004 | 0.217 (3) | 0.052 (2) | 0.376 (3) |
| Jeff010 | 0.007 (2) | 0.468 (3) | 0.176 (3) |
| Jeff011 | 0.288 (5) | 0.776 (7) | 0.629 (5) |
| Jeff029 | 0.502 (3) | 0.661 (7) | 0.236 (3) |
| Jeff036 | 0.440 (5) | 0.577 (5) | 0.214 (4) |
| Jeff058 | 0.049 (4) | 0.513 (4) | 0.571 (3) |
| Jeff059 | 0.014 (2) | 0.644 (4) | 0.479 (3) |
| Jeff067 | 0.007 (2) | 0.438 (2) | 0 (1) |
| Jeff069 | 0.438 (2) | 0.245 (2) | 0.026 (2) |
| Jeff074 | 0.022 (3) | 0.128 (4) | 0.062 ()3 |

**Figure S1** Diversity at microsatellite (PCoA scatterplot) and chloroplast sequence level (each color represents a different haplotype) for samples from the main study site (circles), across Germany and other countries (diamonds).


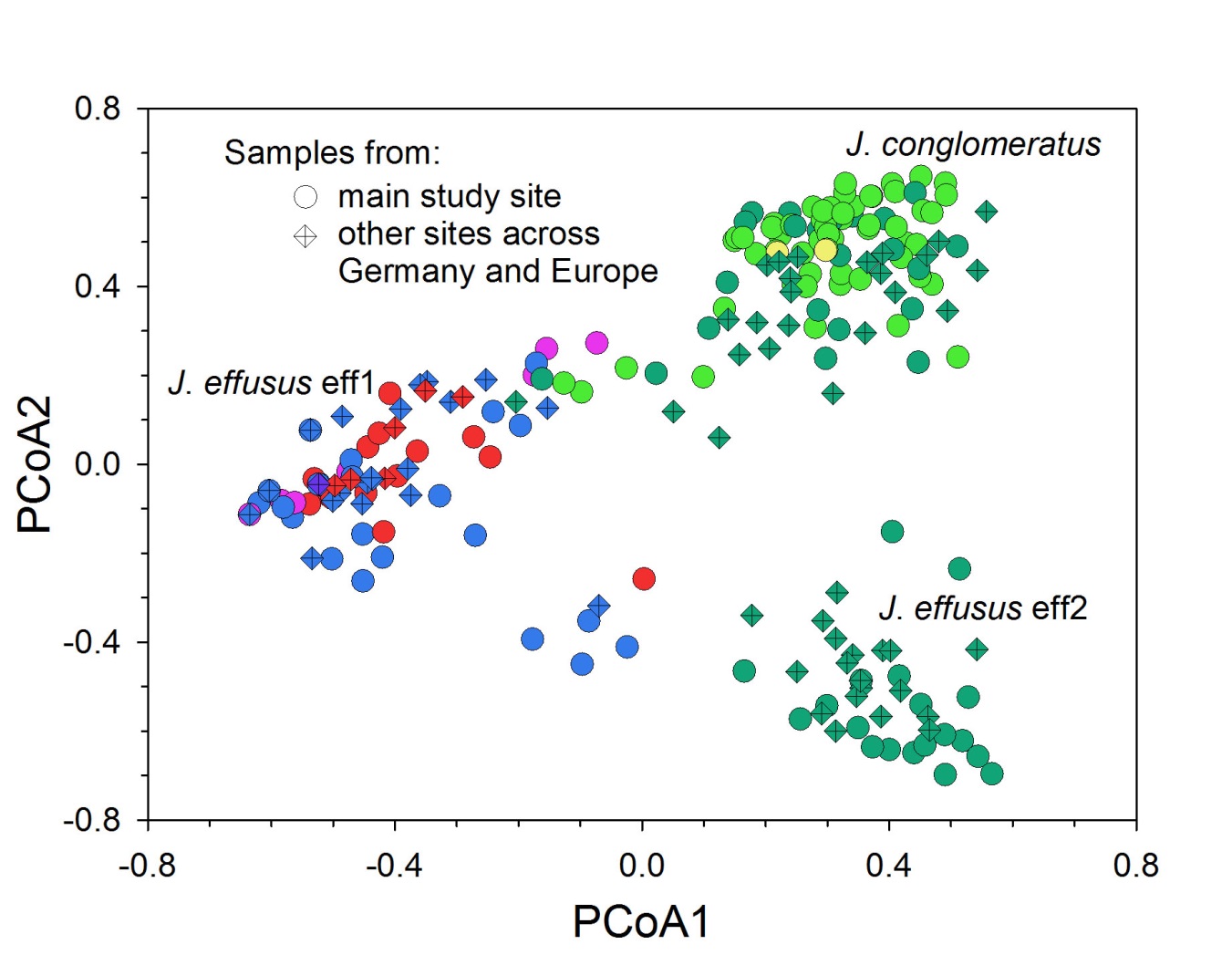


**Figure S2** STRUCTURE summary statistics for different number of clusters *K*. Triangles show the change in mean posterior probability of the data, mean L(K), for increasing *K* (left axis). Circles represent Δ*K* values (right axis).

**Figure S3** Posterior probabilities for different genotypic frequency classes displayed for 25 hybrid individuals based on analyses with NewHybrids. The first 17 individuals were identified as hybrids between *J*. *conglomeratus* (cong) and *J*. *effusus* 1 (eff1), the next two as hybrids between *J*. *conglomeratus* and *J*. *effusus* 2 (eff2), and the last five as hybrids between both *J*. *effusus* groups.

**Figure S4** Spatial distribution of samples at the main study site. Genetically defined pure groups are colored: *Juncus conglomeratus* (cong, red), *J*. *effusus* 1 (eff1, green), *J*. *effusus* 2 (eff2, blue). Gray colors represent genetically identified hybrids (dark gray *J*. *conglomeratus* x *J*. *effusus*, light gray *J*. *effusus eff1* x *eff2*).

**Figure S5** Overlap in flowering between *J*. *effusus* lineages eff1 (N = 23, green) and eff2 (N= 17, blue). Flowering was assessed per individual and as the number of inflorescences with open flowers.


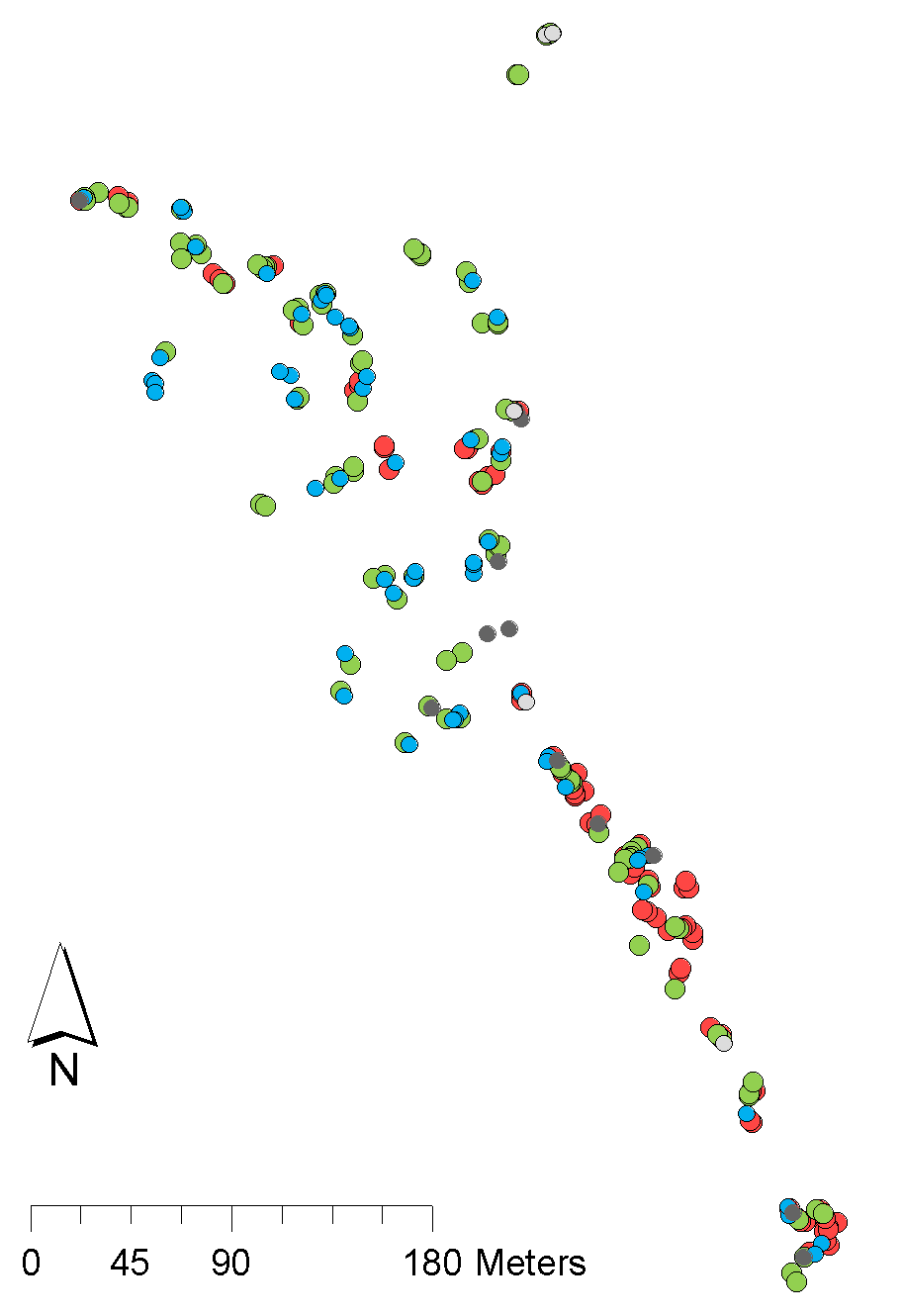

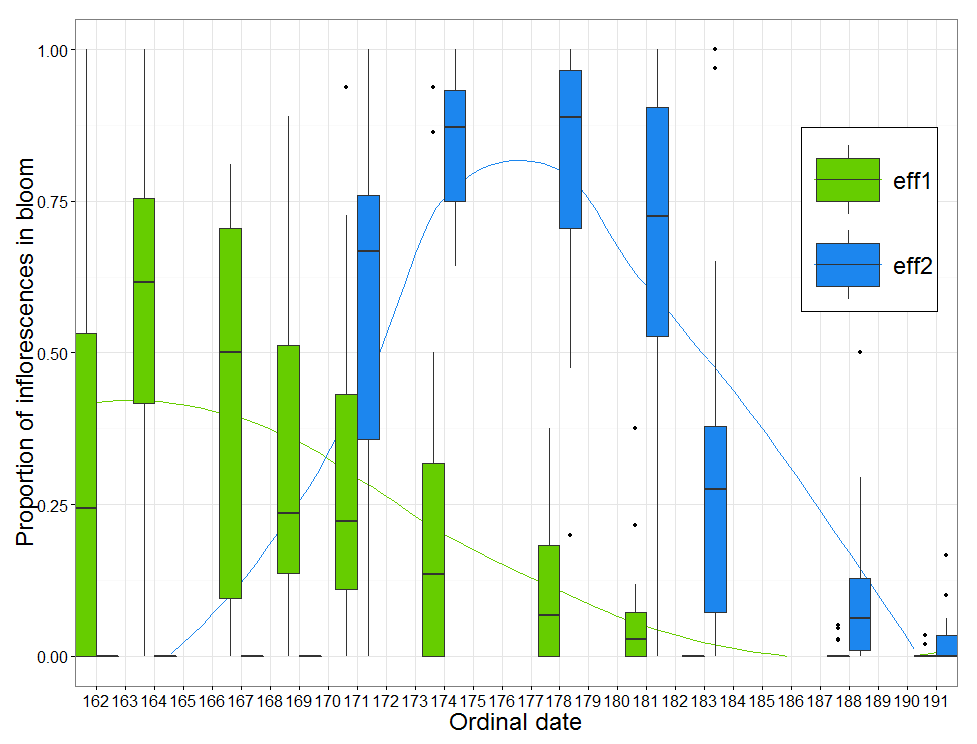


**Figure S6** Scatterplot showing the relation between date of first flowering and predispersal seed predation for pure groups: *Juncus conglomeratus* (cong, red), *J*. *effusus* 1 (eff1, green), *J*. *effusus* 2 (eff2, blue), and hybrids: *J*. *conglomeratus* x *J*. *effusus* (hybce, dark gray) and *J*. *effusus eff1* x *eff2* (hybee, light gray).

**Literature cited**


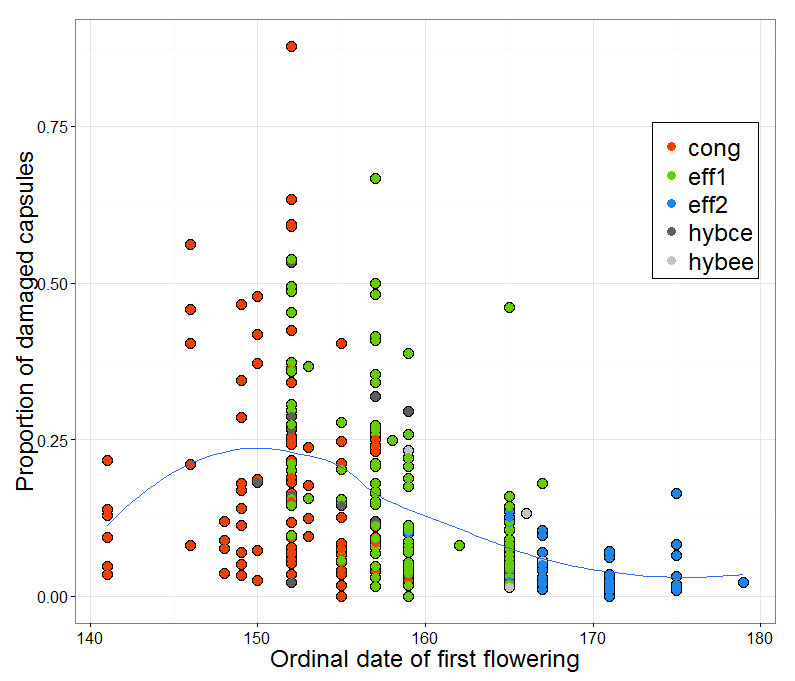


Anderson, E. C. and E. A. Thompson. 2002. A model-based method for identifying species hybrids using multilocus genetic data. Genetics 160:1217-1229.

Ehrich, D., M. Gaudeul, A. Assefa, M. A. Koch, K. Mummenhoff, S. Nemomissa, I. Consortium, and C. Brochmann. 2007. Genetic consequences of Pleistocene range shifts: contrast between the Arctic, the Alps and the East African mountains. Mol. Ecol. 16:2542-2559.

Evanno, G., S. Regnaut, and J. Goudet. 2005. Detecting the number of clusters of individuals using the software STRUCTURE: a simulation study. Mol. Ecol. 14:2611-2620.

Falush, D., M. Stephens, and J. K. Pritchard. 2003. Inference of population structure using multilocus genotype data: Linked loci and correlated allele frequencies. Genetics 164:1567-1587.

Falush, D., M. Stephens, and J. K. Pritchard. 2007. Inference of population structure using multilocus genotype data: dominant markers and null alleles. Mol. Ecol. Notes 7:574-578.

Michalski, S. G. and W. Durka. 2012. Identification and characterization of microsatellite loci in the rush *Juncus effusus* (Juncaceae). Am. J. Bot. 99:e53-e55.

Nei, M. 1987. Molecular evolutionary genetics. Columbia University Press, New York.

Nordborg, M., T. T. Hu, Y. Ishino, J. Jhaveri, C. Toomajian, H. G. Zheng, E. Bakker, P. Calabrese, J. Gladstone, R. Goyal, M. Jakobsson, S. Kim, Y. Morozov, B. Padhukasahasram, V. Plagnol, N. A. Rosenberg, C. Shah, J. D. Wall, J. Wang, K. Y. Zhao, T. Kalbfleisch, V. Schulz, M. Kreitman, and J. Bergelson. 2005. The pattern of polymorphism in *Arabidopsis thaliana*. PLoS Biol. 3:1289-1299.

Pritchard, J. K., M. Stephens, and P. Donnelly. 2000. Inference of population structure using multilocus genotype data. Genetics 155:945-959.

Scarcelli, N., A. Barnaud, W. Eiserhardt, U. A. Treier, M. Seveno, A. d'Anfray, Y. Vigouroux, and J. C. Pintaud. 2011. A Set of 100 chloroplast DNA primer pairs to study population genetics and phylogeny in Monocotyledons. PLoS One 6:e19954.
